# Supplementary figures and images for: Faster Growth Enhances Low Carbon Fuel and Chemical Production Through Gas Fermentation
Source: Front Bioeng Biotechnol. 2022 Apr 12;10:879578. doi: 10.3389/fbioe.2022.879578 (PMC9039284; doi:10.3389/fbioe.2022.879578)

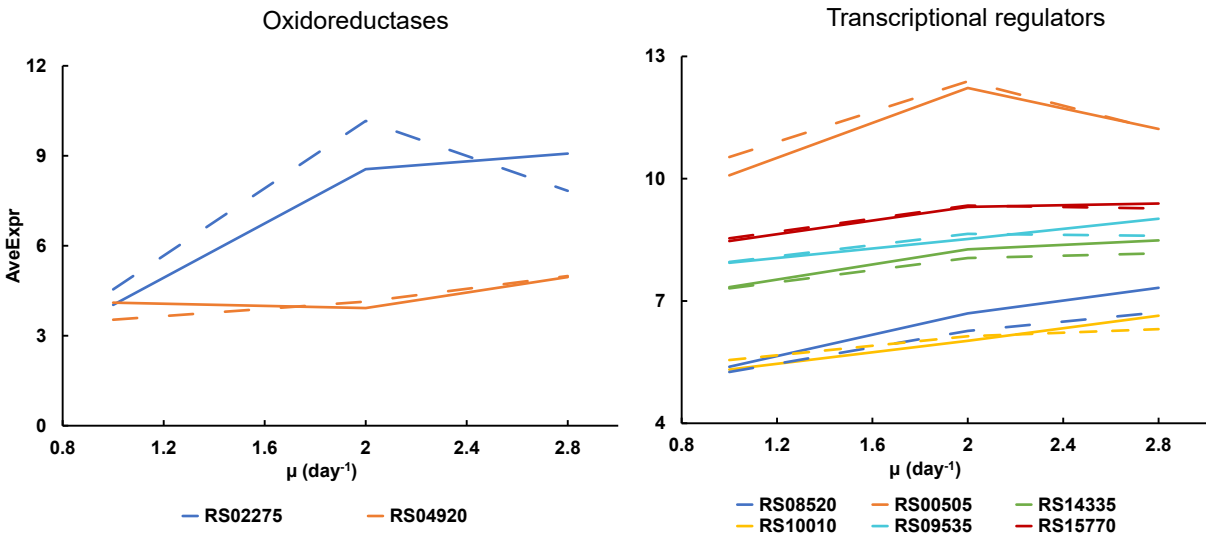

Supplement: Supplementary file 2 [file Image2.PDF]
