# Supplementary material for: Faster Growth Enhances Low Carbon Fuel and Chemical Production Through Gas Fermentation
Source: Front Bioeng Biotechnol. 2022 Apr 12;10:879578. doi: 10.3389/fbioe.2022.879578 (PMC9039284; doi:10.3389/fbioe.2022.879578)
Supplement: Supplementary file 3 [file Image1.PDF]

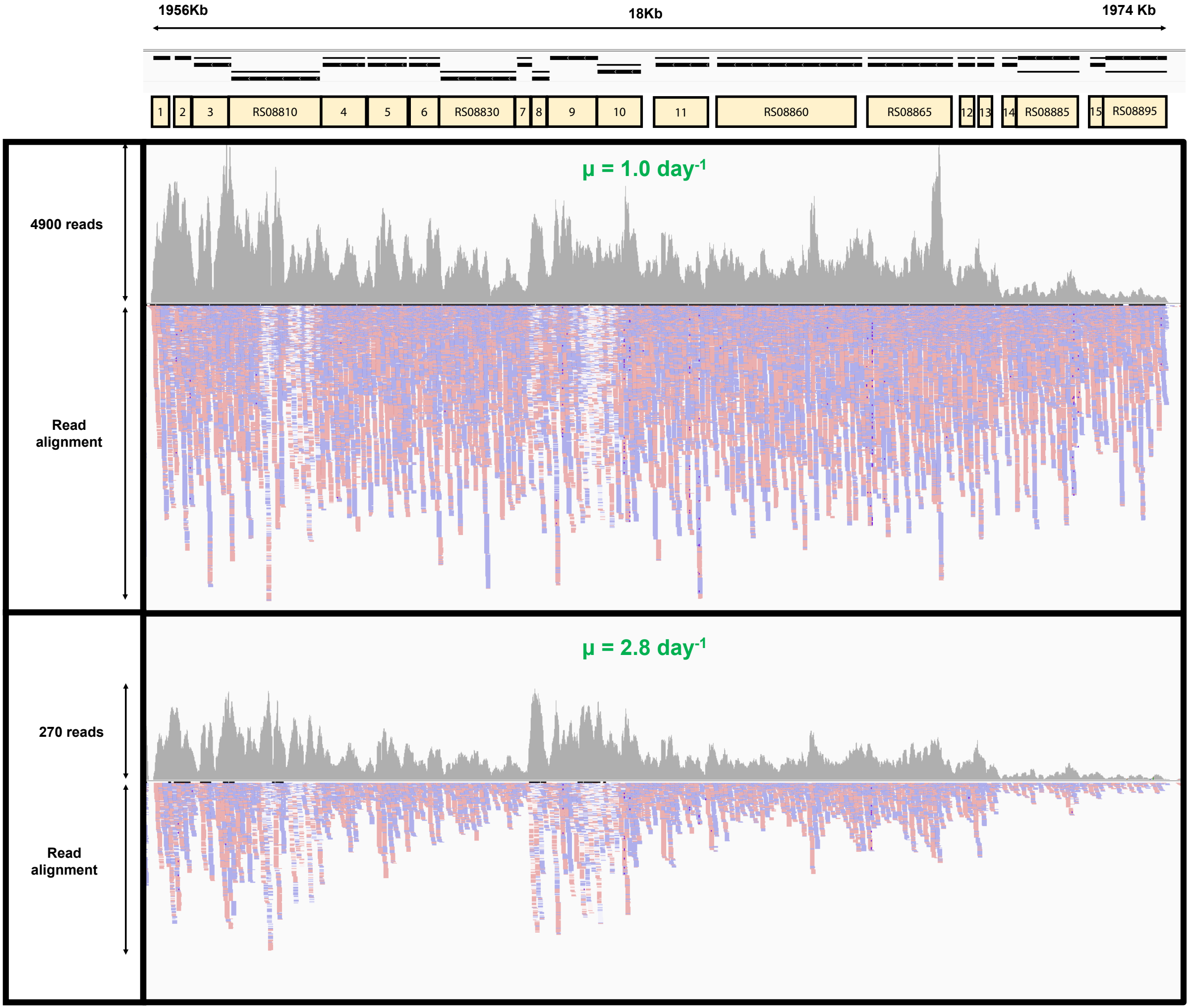

Figure S1 Faster growth leads to strong repression of a cluster of 21 genes (RS08795–08895) linked to bacterial microcompartments (BMCs). Grey upper part shows the wiggle plot of the sequence read coverage. Blue-pink lower part shows mapping of aligned reads where pink indicates (+) and blue (-) strands. Numbers inside yellow boxes above wiggle plot denote gene IDs: 1–3 (RS08795–RS08805), 4–6 (RS08815–RS08825), 7–11 (RS08825–RS08855), 12–14 (RS08870 RS0880), and 15 (RS08890). *μ*, specific growth rate. Gene IDs are preceded with CAETHG_.
